# Bioinspired Nanoplatforms: Polydopamine and Exosomes for Targeted Antimicrobial Therapy

**DOI:** 10.3390/polym17121670

**Published:** 2025-06-16

**Authors:** Barathan Muttiah, Alfizah Hanafiah

**Affiliations:** 1 Department of Medical Microbiology and Immunology, Faculty of Medicine, Universiti Kebangsaan Malaysia, Cheras, Kuala Lumpur 56000, Malaysia; 2 GUT Research Group, Universiti Kebangsaan Malaysia, Kuala Lumpur 56000, Malaysia

**Keywords:** polydopamine (PDA), exosomes, antimicrobial resistance (AMR), hybrid nanoplatforms, stimuli-responsive coatings

## Abstract

Global growth in antimicrobial resistance (AMR) has accelerated the need for novel therapy beyond the scope of conventional antibiotics. In the last decade, polydopamine (PDA), a mussel-inspired polymer with redox capability, remarkable adhesion, and biocompatibility, has emerged as a universal antimicrobial coating with widespread uses. At the same time, extracellular vesicles (EVs) and particularly exosomes have gained prominence for their intrinsic cargo delivery and immune-modulating properties. Here, we summarize the synergistic value of PDA and exosome integration into multifunctional antimicrobial nanoplatforms. We discuss the inherent antimicrobial activity of PDA and exosomes; the advantages of PDA coating, including increased exosome stability, ROS generation, and surface functionalization; and current methodologies towards designing PDA-exosome hybrids. This review also mentions other antimicrobial polymers and nanocomposites that may be employed for exosome modification, such as quaternized chitosan, zwitterionic polymers, and polymer–metal composites. Most significant challenges, such as the maintenance of exosome integrity, coating uniformity, biocompatibility, scalability, and immunogenicity, are addressed. Finally, future research directions are highlighted, with emphasis on intelligent, stimulus-responsive coatings, AMP incorporation, and clinical translation. Collectively, this review underscores the promise of PDA-coated exosomes as potential antimicrobial therapeutics against AMR with potential applications in wound healing, implant protection, and targeted infection control.

## 1. Introduction

The global increase in bacterial infections has fueled the emergence of antimicrobial resistance (AMR), a significant public health concern [1]. AMR is predominantly caused by antibiotic misuse and overuse in human medicine, agriculture, and animal farming, creating multidrug-resistant (MDR) bacteria that undermine traditional treatments [2]. In low- and middle-income countries, unlimited access to antibiotics, unwarranted prescribing, and non-compliance further fuel resistance [3]. Bacteria adapt rapidly through genome plasticity, including mutations, horizontal gene transfer, and acquisition of resistance genes, leading to mechanisms such as target site modifications, efflux pumps, and enzymatic inactivation [4]. AMR is not restricted to bacterial infections but also affects viral, fungal, and parasitic infections and is the cause of treatment failures, prolonged hospital stays, and significant economic burdens, with estimates suggesting a global GDP loss of up to 3.5% by 2050 [5].

AMR treatment demands novel therapeutic strategies in addition to traditional antibiotic treatment [6]. Nanotechnology presents a very promising platform to meet this challenge, with particular emphasis on polydopamine (PDA)-based nanomaterials. PDA has become of greater concern due to its unique physicochemical characteristics, such as strong adhesive property, easy functionalization, high biocompatibility, and intrinsic antibacterial activity [7,8]. It has demonstrated versatility in the synthesis of multifunctional nanomaterials for biomedical applications, mainly in preventing infection, modulating surfaces, and drug delivery.

PDA-modified nanoparticles exhibit enhanced antibacterial performance through multiple mechanisms, including the generation of reactive oxygen species (ROS), inhibition of biofilms, and improved photothermal conversion efficiency [9]. With metal agents like silver nanoparticles, PDA coatings provide a dramatic boost to antibacterial performance and, hence, find applications in wound dressings, wearable devices, and water filtration membranes. These applications underscore the significance of PDA, not just in clinical uses but also in general environmental and industrial uses. Simultaneously, exosomes, spontaneously secreted extracellular vesicles (EVs), have been recognized as promising vectors for antimicrobial therapy. Their intrinsic ability to transport biomolecules, traverse biological barriers, and engage in intercellular communication renders them useful agents for targeted drug delivery and immune modulation in infectious diseases [10].

Here, this review describes the first complete overview integrating PDA and EVs as novel and complementary solutions against AMR. While both have been independently studied, their joint potential, such as PDA as an exosome delivery vehicle, remains unreported. This review creates a bridge between materials science and extracellular vesicle biology and proposes new research directions for multifunctional, next-generation anti-infective therapy. The aim of this review is to critically evaluate and synthesize current knowledge on the antimicrobial properties of PDA and EVs with a specific focus on their roles in combating AMR. This review further aims to explore the potential of integrating PDA and exosome-based strategies to develop multifunctional and synergistic therapeutic platforms for the prevention and treatment of drug-resistant infections. We hypothesize that integrating PDA’s surface-modifying, photothermal, and antimicrobial properties with the immunomodulatory and cargo-delivery capabilities of exosomes represents a promising, underexplored strategy for addressing multidrug-resistant infections.

## 2. Overview of AMR

AMR is a multifaceted global health crisis fueled by a myriad of interconnected human, environmental, and socioeconomic drivers [11]. Antimicrobials are naturally occurring compounds that kill or suppress the growth of microorganisms. They are of the utmost significance in human medicine because they have significantly improved such medical procedures as surgeries and infection control [12]. Aside from human health, antimicrobials also play a very important role in enabling economic livelihoods for millions of animal keepers and farmers worldwide. They preserve animal health, enhance agricultural productivity, and ensure food safety. Access to, as well as the effectiveness of, antimicrobials has widened, and this has ushered in improved agricultural productivity, overall food safety, and animal health [13]. Yet this widespread use of antimicrobials has been accompanied by difficulties, particularly in the form of AMR.

**Figure 1:**
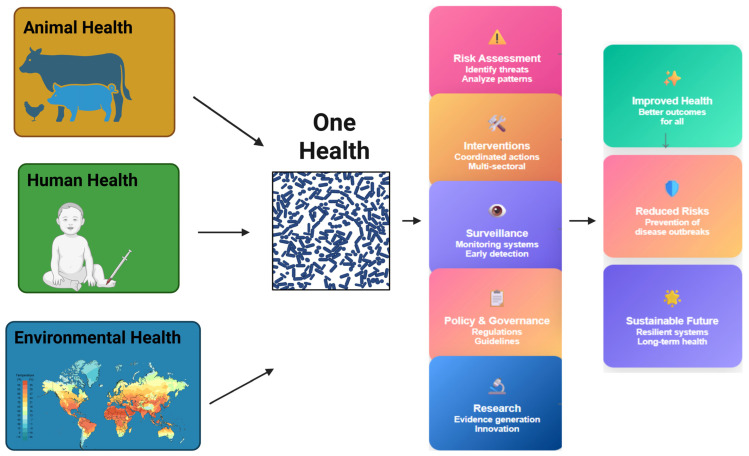
The One Health approach and its interconnected domains influencing antimicrobial resistance (AMR).

The excessive and inappropriate use of antibiotics in human medicine and agriculture is the primary driver of AMR [14]. Within healthcare, inappropriate prescribing, especially for viral infections, is a significant driver of resistance, particularly where antibiotics can be easily obtained over the counter. In addition, the limited number of new antibiotics in the pipeline, poor diagnostic tests, and inadequate antimicrobial stewardship practices exacerbate the problem [15]. Antibiotics in animal husbandry are not just used for treatment but also for disease prevention, which has led to a rise in resistant bacteria in animals and the environment. Environmental aspects also exacerbate AMR, with untreated wastewater, pharmaceutical manufacturing effluents, and agricultural runoff containing residues of antibiotics all contributing significantly to the dissemination of resistance genes [16]. Further, global human, animal, and commodity travel facilitates the rapid transmission of resistant pathogens across borders [17]. Figure 1 shows the One Health framework, highlighting the complex interconnections between human health (pink), animal health (blue), and environmental health (green). Various drivers contribute to AMR across these sectors, including factors like population movements, diet, food security, antibiotic use, intensive livestock farming, disease vectors, climate change, air and water pollution, biodiversity loss, and social determinants of health. The center emphasizes the integrated nature of health systems and the need for coordinated strategies across disciplines to effectively combat AMR and emerging zoonoses.

### Mechanism of Antibiotic Resistance

The resistance of bacteria to antimicrobials has emerged as a growing issue across the globe, with many sophisticated mechanisms giving immunity to the bacteria against therapeutic antibiotics. Apart from limiting the effectiveness of present-day antibiotics, these mechanisms are also responsible for triggering the rampant emergence and spreading of resistance that pose severe health hazards to humanity [18].

Efflux pumps are membrane-embedded transport proteins that play a major role in antibiotic resistance in bacteria by actively pumping a wide range of toxic compounds, including antibiotics, out of the bacterial cell [19]. Efflux pumps are generally classified as either drug-specific or multidrug resistance pumps. Drug-specific efflux pumps target particular classes of antibiotics, while multidrug efflux pumps can extrude a wide range of drugs, leading to cross-resistance to several antibiotic drug classes [20]. These are found in Gram-positive and Gram-negative bacteria and belong to six big families based on their energy source and organization: ATP-binding cassette (ABC) superfamily, major facilitator superfamily (MFS), multidrug and toxic compound extrusion (MATE), resistance–nodulation–division (RND) family, small multidrug resistance (SMR) family, and proteobacterial antimicrobial compound efflux (PACE) family [21,22,23]. Among them, the RND family is particularly important in Gram-negative bacteria. Efflux pumps not only confer natural resistance through chromosomally controlled genes but also contribute to acquired resistance, in which their expression is increased as a result of mutations within the genes or regulatory pathway modulation. This increase may be brought about by environmental stress or antibiotic exposure, leading to cross-resistance, whereby one pump exports not only antibiotics but also biocides, dyes, and detergents [24]. Moreover, efflux pumps are also implicated in biofilm-associated infections by facilitating the exclusion of antimicrobials into the protective biofilm matrix, hence enabling bacterial persistence and tolerance [25]. Clinically, the overexpression of efflux pumps is also generally observed in multidrug-resistant pathogens such as *Escherichia coli* and *Pseudomonas aeruginosa*. In *E. coli*, the AcrAB-TolC system, a tripartite inner membrane RND-type efflux pump, periplasm, and outer membrane, exports a variety of antibiotics, like fluoroquinolones, chloramphenicol, rifampicin, and tetracycline [26]. The system is normally overexpressed in uropathogenic strains (UPEC), particularly biofilm-forming clinical isolates, resulting in persistent infections. Similarly, in *P. aeruginosa*, the MexAB-OprM efflux pump, AcrAB-TolC homologous, plays a role in resistance to β-lactams, fluoroquinolones, chloramphenicol, and trimethoprim. MexAB-OprM overproduction not only decreases susceptibility to these antibiotics but also to biocides like triclosan, demonstrating the cross-resistance phenomenon [27,28]. Efflux pumps, therefore, present an enormous challenge to effective antimicrobial treatment, not so much by directly mediating resistance but by increasing bacterial survival under antibiotic stress, allowing for the establishment of other forms of resistance mechanisms such as target site mutations [29]. Their involvement in vital bacterial processes such as virulence, stress response, and biofilm formation attests to their importance beyond antibiotic resistance. Although the inhibition of efflux pumps by efflux pump inhibitors (EPIs) is a tempting therapeutic approach to restore antibiotic activity, challenges such as substrate redundancy, pump multiplicity, and potential EPI toxicity have forestalled clinical success [30]. Yet, awareness of the molecular mechanisms and control networks of efflux pumps is essential in coming up with effective steps to combat multidrug-resistant bacterial infections [31].

Bacteria produce many enzymes that render antibiotics ineffective through the chemical breakdown or alteration of the molecules of the antibiotics, and this is a major mechanism by which they develop resistance to treatment and become resistant [32]. Some of these include the most widely recognized, β-lactamases, which hydrolyze the β-lactam ring, a structural element most crucial to the function of β-lactam antibiotics, such as penicillins, cephalosporins, carbapenems, and monobactams, rendering them ineffective [33]. These enzymes consist of four molecular classes (A, B, C, and D), with classes A, C, and D being serine hydrolases and class B being a zinc-dependent metallo-β-lactamase. Aminoglycoside-modifying enzymes (AMEs) are another well-known group, inactivating aminoglycoside antibiotics through acetylation, phosphorylation, or adenylation, blocking their binding to bacteria’s ribosomes, an example being aminoglycoside-6′-N-acetyltransferase-Ib [AAC(6′)-Ib] [34]. Similarly, macrolide esterases hydrolyze macrolides and tetracycline destructases such as the flavin-containing monooxygenase TetX oxidize tetracyclines, including tigecycline, to induce antibiotic degradation [35]. Rifamycin-modifying enzymes such as ADP-ribosyltransferases, kinases, and glycosyltransferases modify critical hydroxyl functional groups on rifamycins, inhibiting them from binding to RNA polymerase [36]. In addition to these, there are other enzyme systems, such as phosphotransferases, nucleotidyltransferases, methyltransferases, oxidoreductases, and lyases, that chemically alter other antibiotics like chloramphenicol, streptogramins, and fosfomycin through hydrolysis, group transfer, or redox reactions, ultimately stopping their activity [37]. The resistance enzymes are usually regulated by genes located on mobile genetic elements such as plasmids, transposons, and integrons, enabling them to be horizontally transferred between bacterial populations and increase the spread of multidrug resistance [38]. The enzymatic inactivation of antibiotics is a clinically significant issue, undermining therapy and leaving narrow therapeutic windows for most infections, including those caused by multidrug-resistant pathogens. Understanding these enzymatic activities is essential to the design of inhibitors, novel antibiotics, and other forms of therapy to address the increasing threat of antibiotic resistance.

Meanwhile, target site alteration is one of the most widespread bacterial mechanisms of resistance to antibiotics, wherein bacteria alter the molecular target (protein or nucleic acid) upon which antibiotics are designed to operate, thereby minimizing or abolishing drug action while preserving essential cellular functions [39]. Such resistance can arise from the spontaneous mutation of target-encoding genes, enzymatic modification of the target site, or acquisition of foreign target proteins via horizontal gene transfer. For instance, rpoB gene point mutations alter the RNA polymerase’s β-subunit to confer resistance to rifamycins in *Mycobacterium tuberculosis* [40], while mutations in gyrA and parC genes that affect DNA gyrase and topoisomerase IV result in resistance to fluoroquinolones in a variety of bacterial species [41]. In addition, enzymatic alterations, such as the methylation of 23S rRNA by methyltransferases, prevents MLS antibiotics binding, while the methylation of 16S rRNA confers resistance to aminoglycosides [42]. Bacteria also evade drug action by acquiring low-antibiotic-affinity alternative target proteins such as *Staphylococcus aureus*, which acquires the mecA gene coding for PBP2a, a penicillin-binding protein with low β-lactam affinity, resulting in methicillin-resistant *S. aureus* (MRSA) [43], while *Streptococcus pneumoniae* synthesizes altered PBPs that render bacteria resistant to β-lactams [44]. Target protection proteins are also synthesized in certain situations, physically preventing antibiotic targets from interaction with drugs but not altering the target molecule itself. Such alterations have clinical relevance since they prevent or eliminate drug binding but often do not impair the natural function of the target molecule, thus allowing bacteria to grow and thrive despite treatment [45]. Because such mechanisms cross many antibiotic classes and bacterial pathogens, target site modification is the most important cause of therapeutic failure and of the global phenomenon of multidrug-resistant infection.

The reduced membrane permeability of Gram-negative bacteria is a consequence of antibiotic resistance. This specific mechanism relates to the unique features of the bacteria’s outer membrane, which acts as a gatekeeper and hinders the free flow of antibiotics into the cell [46]. The membrane contains a phospholipid bilayer with an inner leaflet of phospholipids and an outer leaflet of lipopolysaccharide (LPS), a structural component of Gram-negative bacteria, which creates a hydrophobic funnel that is further reinforced by Mg^2+^ or Ca^2+^ cations [47]. All these factors strongly limit the ability of hydrophobic antibiotics to break through the bacterial defenses. Hydrophilic antibiotics, on the other hand, such as chloramphenicol, fluoroquinolones, and β-lactams, depend on porins to transport them across the membrane [48]. Porins are β-barrel proteins that act as size-selective channels that create aqueous pathways. The blockade arises when bacteria exhibit reduced expression of porins, undergo genomic alterations to porin coding sequences, or substitute major porins with narrower and less functional substitutes [49]. An example of this is shown with *P. aeruginosa’s* resistance to imipenem, which stems from a lack or alteration of the OprD porin, while *Klebsiella pneumoniae* and other *Enterobacteriaceae* display mutations in porins like OmpK36, contributing to reduced β-lactam susceptibility. In addition, *E. coli* mutants lacking the OmpF porin show increased resistance to multiple antibiotics including β-lactams and quinolones. Such variations altered by porins can grant resistance against not just one but multiple classes of antibiotics. Such modifications are more frequent alongside other mechanisms [50,51].

Biofilm formation is a key factor in antibiotic resistance by creating a complex and sheltered microenvironment that shields bacterial communities against antimicrobial substances [52]. Central to such resistance is the extracellular polymeric substance (EPS), matrix made up of exopolysaccharides, extracellular DNA (eDNA), and proteins, that acts as a barrier to the permeation of antibiotics and facilitates enzymatic antibiotic inactivation, such as with β-lactamases produced by *K. pneumoniae* and *P. aeruginosa* [53]. These multilayered defenses make biofilm-associated bacteria 10–1000-fold more antibiotic resistant than planktonic counterparts. Clinically, this is evident in *Staphylococcus epidermidis*, which, while vancomycin is susceptible in a planktonic culture, is reportedly up to 75% resistant in biofilms; *K. pneumoniae*, which is more resistant in biofilms [54]; *P. aeruginosa*, which causes chronic infections in patients with cystic fibrosis; and *Staphylococcus aureus* and *Coagulase-negative staphylococci* (CoNS), which have the capability to colonize medical devices [55]. These illustrations indicate the challenge of the eradication of biofilm-related infections due to the synergy between structural, enzymatic, metabolic, and genetic mechanisms of resistance.

Immune evasion by bacteria is a key process in the development and maintenance of antibiotic resistance through the facilitation of the pathogen evasion of host immune mechanisms, prolongation of infection, and time for the establishment and maintenance of resistance traits [56]. Bacteria achieve this in a number of different ways, such as biofilm formation that shields them from both immune effector molecules and antibiotics, inducing chronic infection in which reduced bacterial growth rate persister cells and reduced antibiotic penetration enhance survival [57,58]. They also regulate host immunity by inducing anti-inflammatory cytokines such as IL-10 and TGF-β, which suppress immune cell activation and slow bacterial killing, indirectly prolonging the window for the emergence of resistance [58]. Intracellular life within macrophages or other host cell types provides a second refuge, protecting bacteria from immune attacks and limiting antibiotic access. Bacteria also modify their surface structures such as LPS modifications, capsule formation, and antigenic variation to evade immune detection by immune cells and antibodies [59]. Enzymatic functions also come into play, with some pathogens secreting proteases that degrade antibodies or complement components, preventing critical immune functions. Clinically significant examples include *M. tuberculosis*, which inhibits immune responses to persist in the host and survive antibiotic treatment [60]; *P. aeruginosa* and *S. aureus*, which form immune-evasive biofilms [61]; *Haemophilus influenzae*, which degrades immunoglobulins in an attempt to evade opsonization [62]; and encapsulated *Neisseria meningitidis* and *E. coli*, which are phagocytosis evading [63,64]. These immune-evasive mechanisms operate in conjunction to create niches in which bacteria persist under immune stress and antibiotic therapy, thereby resulting in treatment failure, reinfection, and the spread of multidrug-resistant strains. Coupled with antimicrobial resistance mechanisms, these evasion mechanisms make infections harder to treat. Table 1 shows the direct resistance mechanisms and facilitating factors caused by bacteria.

Because of the complexity of such mechanisms, combat against bacterial resistance needs to be multifaceted. This means developing new antibiotics that target novel pathways or mechanisms of bacterial survival and using adjunct therapies that may interfere with mechanisms of resistance [65]. In addition, interventions to avert the spread of resistance, such as antimicrobial stewardship programs, infection control, and responsible antibiotic use in agriculture, are essential to curb the incidence of resistant organisms and preserve the effectiveness of available antimicrobial agents [66].

AMR imposes serious clinical and economic costs on healthcare systems and patients. Only in the United States, the annual cost of AMR is USD 55 billion, with USD 20 billion being spent on healthcare and another USD 35 billion being lost in productivity [67]. AMR might be even worse in low-income nations, where it would raise poverty levels and cut 1% of worldwide GDP by 2050, with poorer nations losing as much as 7% [68]. The World Bank estimated that drug-resistant tuberculosis alone would cost the world USD 16.7 trillion by 2007 [69]. These economic burdens are further increased by the ongoing COVID-19 pandemic, where there has been increased use of broad-spectrum antibiotics despite the fact that the prevalence of bacterial and fungal co-infections in COVID-19 patients is low [70].

The AMR crisis requires a One Health approach, integrating efforts across human health, veterinary medicine, agriculture, and the environment [71]. Key strategies include enhanced surveillance, public education on responsible antibiotic use, and policy reforms to regulate antibiotic prescription and usage in both healthcare and agriculture. A global, collaborative effort is essential to address AMR and preserve the effectiveness of antibiotics [72], alongside the exploration of novel therapeutic agents such as nanoparticles, phage therapy, and antimicrobial peptides [73]. Recent advancements have expanded the therapeutic landscape to include exosome-based drug delivery systems [74], CRISPR-Cas antimicrobials targeting resistance genes [75], quorum-sensing inhibitors [76] that block bacterial communication, and microbiota-based interventions like probiotics and fecal microbiota transplantation. Additionally, synthetic polymers, including cationic polymers, polymer–drug conjugates, dendrimers, and stimuli-responsive carriers, offer targeted delivery and enhanced antimicrobial activity [77]. Synthetic biology approaches and engineered probiotics are also being developed to detect and eliminate resistant pathogens, collectively forming a multifaceted arsenal against AMR [78]. Cutting-edge research is vital to identify novel combinations of antibiotics that can outsmart resistance and make last-resort drugs more effective. Table 2 summarizes innovative strategies under investigation or development to address AMR, including their core mechanisms, representative examples, and potential clinical or therapeutic benefits.

## 3. Polydopamine (PDA)

The application of synthetic antimicrobial polymers as implants and medical devices is a developing field, helped by the pressing need to regulate infections that arise with implanted technologies [79]. These infections, typically related to microbial colonization and biofilm maturation, are a key source of medical complications, particularly for immunocompromised subjects or those in need of large-scale device utilization [80]. One of the important methods involves the coating of medical devices using antimicrobial polymers that establish a non-leaching surface that is resistant to microbial growth and adhesion. The surfaces are designed to exert prolonged functionality without releasing toxic materials into neighboring tissues [81]. Additionally, nanocomposite addition such as silver or zinc oxide nanoparticles to polymer matrices has been shown to improve antimicrobial activity significantly, especially against drug-resistant organisms like MRSA [82]. Advanced synthetic polymers, like electrospun nanofibers, are being engineered not only to exhibit antimicrobial function but also to promote tissue integration and healing [83]. These polymers are currently used in complicated surgical reconstructions, such as those for musculoskeletal injuries, where infection prevention is crucial for reducing implant failure [84]. Also, antimicrobial polymers have found their application in other devices, from dental implants to prosthetics, vascular grafts, and pacemaker electrodes, resulting in improved biocompatibility, patient comfort, and device longevity [85]. In summary, synthetic antimicrobial polymers are a revolutionary new technology for medical devices. They offer the double benefits of infection prevention and improved device performance. Among the emerging materials used to enhance the antimicrobial functionality of synthetic polymers, PDA has gained significant attention due to its unique physicochemical properties. Inspired by mussel-adhesive proteins, PDA offers a versatile platform for surface functionalization, enabling the development of advanced antimicrobial coatings [86]. Its ability to bind to a wide range of substrates and coordinate with metal ions makes it an ideal candidate for creating multifunctional, infection-resistant medical devices [87].

### 3.1. Physicochemical Characteristics of PDA

PDA is an artificial biopolymer inspired by mussel-adhesive protein, which is generated via the alkaline oxidative polymerization of dopamine. It contains abundant catechol, amine, and imine groups in its structure, thus having high chemical reactivity, and is a very promising material for surface functionalization, drug encapsulation, and the construction of nanoplatforms for biomedical and environmental uses [88,89]. PDA has been widely regarded as a multifunctional material due to its unique physicochemical properties, including strong adhesion, tunable reactivity, and excellent biocompatibility [90].

One of the most typical properties of PDA is its strong and universal adhesion. Inspired by mussel adhesion, PDA strongly adheres to a wide range of surfaces, including metals, ceramics, polymers, and biological tissues, through covalent and non-covalent interactions such as hydrogen bonding and π–π stacking [91,92,93,94,95]. Notably, this adhesive property is maintained in aqueous conditions, making PDA highly suitable for biomedical applications in wet interfaces [96]. Apart from its structural role, PDA also adds antimicrobial function through membrane disruption and oxidative stress.

PDA is biocompatible in nature and preserves cell viability, which is the foundation of its growing use in drug delivery systems, implant surface coatings, biosensors, and tissue engineering scaffolds [97]. Its hydrophilicity, which is imparted by polar functional groups, facilitates aqueous dispersibility, homogeneous film formation, and enhanced cell adhesion and proliferation [98]. Morphologically, PDA can be tailored into a range of nanostructures, such as nanoparticles, nanocapsules, ultrathin films, and core–shell structures, permitting tailoring for applications like drug targeting, imaging, and catalysis [99]. While bulk PDA is water-insoluble, PDA nanoparticles are soluble in organic solvents like DMSO, DMF, and ethanol. Surface modification like PEGylation also improves their solubility and colloidal stability under physiological and harsh conditions [100].

Chemically, the reactivity of PDA relies on its catechol, amine, and imine groups that readily conjugate with nucleophilic molecules, enabling surface engineering, biosensing, and biomolecule immobilization [101]. The catechol groups can oxidize to quinones, enabling metal chelation, redox activity, and covalent cross-linking that are essential for bioactive loading and catalytic activity [102]. PDA can also generate reactive oxygen species (ROS), including superoxide and hydrogen peroxide, under specific conditions. This is utilized for antimicrobial and anticancer therapy but must be stringently controlled to prevent off-target cytotoxicity [103]. PDA is also a very good platform for the conjugation of secondary functional groups like thiols, hydroxyls, and amines via Michael addition or Schiff–base reactions. It also stabilizes various metal nanoparticles, broadening its application in catalysis, magnetic separation, and sensor technologies [104].

PDA is electrochemically superior in its redox behavior, making it suitable for applications in energy storage devices, biosensors, and electrocatalysis [105]. It acts as an electron donor, especially under metal coordination or near-infrared (NIR) light irradiation, leading to the production of ROS, which destroys bacterial membranes and initiates antimicrobial activity. PDA is also a very effective photothermal agent capable of converting NIR light to heat with high efficiency, which is at the basis of its use in photothermal therapy (PTT) of infections and cancer [106]. Last but not least, PDA also possesses catalytic activity, both by itself and synergistically in combination with nanoparticles such as silver, which further increases its antibacterial activity [107]. Table 3 outlines the multifunctional characteristics of PDA, including its adhesive behavior, chemical reactivity, biocompatibility, and unique structural, optical, and electrochemical properties.

### 3.2. Antimicrobial Activity of Polydopamine

PDA has antimicrobial activity, strongly credited to the occurrence of an atypical combination of functional groups such as catechol, amine, and imine coupled with its strong redox properties [108]. The catechol moieties in PDA will oxidize spontaneously under aerobic conditions to form semiquinone and quinone intermediates while, at the same time, generating ROS in the forms of H_2_O_2_ and superoxide anions (O_2_^−^). These ROS cause bacterial membrane disruption, protein oxidation, and nucleic acid damage, which ultimately lead to the death of bacterial cells [109]. Meanwhile, protonated amine groups electrostatically interact with negatively charged bacterial membranes and contribute to membrane disintegration and cell lysis [110]. While imine groups do not directly engage in antimicrobial activity, they enhance the overall chemical reactivity of PDA and enable functionalization with a range of antimicrobial agents [111]. The antimicrobial activity of PDA is also affected by its physical properties. Roughened PDA (rPDA) coatings enhance the surface contact with bacteria and facilitate mechanical and oxidative membrane destruction. It has been demonstrated through studies that rPDA coatings have almost full antibacterial activity against *S. aureus*, *E. coli*, and *P. aeruginosa*, while smooth PDA (sPDA) coatings show low efficacy [108,112]. In addition, PDA’s catechol moieties possess strong metal-chelating properties that allow for the formation of PDA–metal hybrids, particularly with silver ions (Ag^+^). The hybrids stabilize silver nanoparticles and enhance ROS production, which causes prolonged and synergistic bactericidal activities [113]. Thin films of PDA-Ag nanoparticles, even polyethylene glycol-functionalized, exhibited antimicrobial activity for several days and are, therefore, suitable for biomedical applications [114]. PDA’s redox duality (pro-oxidant and antioxidant) allows it to respond to environmental signals such as pH, light, and metal ion concentrations, which modulates ROS production contextually and presumably lowers resistance in bacteria [113,114,115,116]. Additionally, PDA’s excellent UV to near-infrared (NIR) light absorption enables photothermal therapy (PTT). When exposed to NIR light, PDA photoconverts to heat (>50 °C), inducing localized bacterial killing by protein denaturation and membrane disruption without harming host tissues [117]. PDA-based systems for PTT are PDA-ZIF-8 nanostructures for biofilm removal, PDA-based microneedles for skin infections [118], and PDA-loaded multifunctional hydrogels with imaging and infection control functions [119]. PDA has also been used in photodynamic therapy (PDT) with its redox-active nature allowing light-induced ROS generation for antibacterial application [120,121]. PDA composites, such as PDA–metal–organic frameworks (MOFs), PDA–curcumin hybrids, and PDA/Fe_3_O_4_ nanozymes, also illustrate their application in ROS-mediated cytotoxicity towards bacteria [122,123]. Aside from ROS-mediated mechanisms, PDA coatings can be chemically modified to yield N-halamine-based antibacterial coatings. Halogenated amine groups on the surface of PDA release oxidative halide species (e.g., Cl^+^), which destroy bacterial proteins and enzymes. These surfaces exhibit outstanding durability and repeated antibacterial activity, such as in the case of PDA/PEI-chlorinated films and other halamine-functionalized coatings [124,125,126,127].

### 3.3. Synergistic Antimicrobial Systems Based on Polydopamine

The strong surface adhesion, reactive catechol and amine moieties, and mild synthesis conditions of PDA make it a great scaffold for multifunctional antimicrobial platforms [128]. Notably, PDA coatings allow for the covalent or non-covalent immobilization of antimicrobial peptides (AMPs), which allows for localized and prolonged antibacterial activity and minimal systemic toxicity [129,130]. For instance, KR-12, a cationic fragment of AMP from human cathelicidin LL-37, has been grafted onto PDA-coated implants, providing antibacterial shielding and improved osteogenic differentiation [130]. Similarly, a PDA-lipopeptide (SL1.15) coating designed for Ultrathane catheters potently inhibited *S. aureus* and *E. coli* biofilm growth (>85%) and bacterial attachment (>90%) without toxicity to human cells [131]. CWR11-grafted PDA-coated polydimethylsiloxane (PDMS) catheters displayed sustained antimicrobial activity against CAUTI-related pathogens for 21 days with minimal cytotoxicity [132]. Peptidomimetic antimicrobials such as Melimine, Mel4, and RK758 were immobilized on PDA-coated surfaces with ease using a one-pot method, which showed high activity against *S. aureus*, *E. coli*, and *P. aeruginosa* [133]. Moreover, PDA nanoparticles with polyarginine modification also showed high antimicrobial activity against *S. aureus* through electrostatic membrane interaction [134]. PDA conjugates of LL-37 and its analogs, such as PGLa, have also shown dual anti-inflammatory and wound-healing activity [135]. In addition to peptides, PDA can enable the introduction of antibiotics and metal nanoparticles. For example, PDA-graphene oxide (GO) composite films with tetracycline and green-synthesized Ag nanoparticles significantly suppressed *S. aureus* and *E. coli* biofilms by >1000-fold. Antibacterial properties were estimated via Kirby–Bauer diffusion assays, colony forming unit (CFU) counts, live/dead staining, and biofilm imaging [136]. A comparative study of PDA-based Ag and gentamicin (Gen) coatings, Ag@Gen/PDA (in situ loading) and Ag/Gen@PDA (physical adsorption) verified that Ag@Gen/PDA released antimicrobial agents at a lower rate, with sustained release behavior over 30 days and higher long-term inhibition of bacteria [137]. PDA nanoparticles have even been used for combination photothermal–antibacterial treatments. For instance, PDA-PEI nanoparticles with Polymyxin B showed more than 96% and 99.7% inhibition of bacteria under NIR light against *S. aureus* and *E. coli*, respectively, and an enhancement in wound healing through fibroblast activation and angiogenesis in vivo [138]. A few other PDA-based hybrid systems include PDA-Au nanoparticle systems, where AuNPs have been employed to facilitate the entry of PDA into bacterial cells, and PDA improves the stability and dispersibility of AuNPs [127]. PDA coatings have also immobilized antimicrobial enzymes like lysostaphin effectively, without losing their bioactivity and inhibiting bacterial colonization [139]. Lastly, PDA-functionalized titanium dioxide (TiO_2_) surfaces combine PDA’s adhesive and stabilizing properties with TiO_2_’s photocatalytic ROS-generating activity under UV light to enhance antimicrobial activity for implantable devices [140]. In total, these findings demonstrate PDA’s impressive versatility in the development of next-generation antimicrobial coatings through synergistic interactions with peptides, antibiotics, nanoparticles, enzymes, and photocatalysts. Table 4 outlines the major functional strategies of PDA in antimicrobial applications, highlighting its redox activity, surface chemistry, and ability to serve as a scaffold for synergistic systems.

## 4. Extracellular Vesicle-Based Therapeutics

### 4.1. Biology and Function of Extracellular Vesicles

Extracellular vesicles (EVs) are lipid bilayer-enclosed nanoscale particles secreted by nearly all living cells and are the agents of intercellular communication. The vesicles regulate critical biological processes, such as immune modulation, angiogenesis, fibrosis, and metastasis [141]. EV cargo, comprising nucleic acids, proteins, lipids, metabolites, and even organelles, comprises potent molecular vehicles of information that reflect the physiological state of cells, tissues, and organs [142]. Exosome formation is a regulated, multi-step process that originates from the endosomal route to result in the secretion of sEVs, ranging in size typically between 30 and 150 nm in diameter [143]. According to the new MISEV2023 guidelines [144], EV subtype characterization, including exosomes, must be based on multiple, complementary approaches, particularly when their formation is inferred. The process begins with the endocytosis of plasma membrane components and extracellular material by a variety of types of endocytic routes (clathrin- and caveolin-mediated pathways), leading to early endosomes. These are subsequently mature to late endosomes or multivesicular bodies (MVBs), in which invagination of the endosomal membrane leads to the formation of intraluminal vesicles (ILVs) that serve as precursors for exosomes [145]. The formation of ILVs is regulated by both ESCRT-dependent and ESCRT-independent mechanisms. The ESCRT machinery is composed of four protein complexes (ESCRT-0 to -III) and regulatory proteins such as ALIX and TSG101, which are implicated in cargo recognition, membrane budding, and scission [146]. ESCRT-independent mechanisms, on the other hand, employ ceramide-mediated lipid raft microdomains and tetraspanins (CD9, CD63, CD81) to aid in ILV formation and cargo sorting. Selective packaging of cargo proteins, nucleic acids (miRNAs, mRNAs, lncRNAs), lipids, and metabolites into ILVs is regulated by signal motifs, RNA-binding proteins (hnRNPA2B1, YBX1), and post-translational modifications like ubiquitination [147]. When matured, MVBs can either fuse with lysosomes to be degraded or with the plasma membrane to release ILVs as exosomes into the outside environment. Rab GTPases (namely Rab27a/b, Rab11, and Rab35) and SNARE proteins that regulate vesicle docking and membrane fusion regulate this release via exocytosis [148]. Secreted, exosomes then interact with target cells through endocytosis, direct membrane fusion, or receptor–ligand binding, altering varied physiological as well as disease processes [149].

Large extracellular vesicles (large EVs), formerly and still often termed microvesicles, microparticles, or ectosomes, are a clear subtype of EVs distinct from small EVs (e.g., exosomes) [150]. In line with the MISEV2023 guidelines, the focus is now on functional and operational terminology instead of inflexible nomenclature based purely on biogenesis or size unless backed by strong mechanistic data [144]. Large EVs measure 100 nm to >1 µm in diameter and originate from outward budding and fission of the plasma membrane, in contrast to the endosomal origin of small EVs [151]. The biogenesis of large EVs is marked by cytoskeletal reorganization, membrane curvature, and the activities of signaling proteins such as ARF6, RhoA, and calcium-dependent proteases like calpain. As they bud from the cell surface, large EVs will incorporate surface proteins characteristic of the plasma membrane and cytosolic contents, thus reflecting the phenotype and functional status of the parent cell [152]. Because of their plasma membrane derivation, they will also expose phosphatidylserine on their outer leaflet, which can be unveiled by annexin A5 or annexin A1. Other surface markers that are frequently present in large EVs include integrins, selectins, CD40, and ARF6 [153,154]. Table 5 summarizes the key differences between small extracellular vesicles (sEVs), commonly referred to as exosomes, and large extracellular vesicles (large EVs), often termed microvesicles or ectosomes.

### 4.2. EVs in Infectious Diseases

Due to their natural role in intercellular communication and their ability to carry bioactive molecules, exosomes have gained significant attention as a potential tool in addressing one of the world’s most pressing health threats, AMR. EVs offer a novel therapeutic approach, as they can deliver bioactive molecules that regulate immune responses, disrupt bacterial quorum sensing, and deliver antimicrobial agents directly to infected tissues or cells [155].

Bovine milk-derived exosomes have been shown to effectively deliver antibacterial agents, such as isobavachalcone and polymyxin B, exhibiting potent antibacterial activity by eliminating 99% of MDR bacteria within four hours [156]. These exosomes demonstrated near-complete microbial inhibition in fresh orange juice and accelerated wound healing in mouse models, showing their potential for applications in both the food industry and animal health. Meanwhile, exosome-like EVs from *Apis mellifera* honey, known as HEc-EVs, contain antibacterial peptides like MRJP1, defensin-1, and jellein-3, as well as exosomal markers (CD63 and syntenin). These HEc-EVs have demonstrated strong antibacterial and antibiofilm activities, particularly against *Streptococcus mutans* [157]. Exosomes from pasteurized cow’s milk have shown a dose-dependent inhibition of *S. aureus* ATCC 25923, reducing colony forming units (CFUs) and extending lag and generation times [158]. Camel milk-derived exosomes (CM-EXOs) exhibit bacteriostatic effects against Gram-negative bacteria such as *E. coli* but are not effective against Gram-positive bacteria like *S. aureus* [159]. Bovine colostrum-derived exosomes inhibit the growth of *S. aureus* by disrupting oxidative phosphorylation, thereby reducing ATP production and causing bacterial death [160]. Exosomes from MSCs demonstrate antibacterial effects against both Gram-positive and Gram-negative bacteria. These effects are attributed to the secretion of AMPs, including beta-defensin-2, via the TLR-4 signaling pathway [161]. Additionally, MSC-derived exosomes enhance antibacterial defense by boosting the phagocytic activity of immune cells such as macrophages and neutrophils. They indirectly support bacterial clearance by promoting macrophage polarization to an anti-inflammatory, phagocytic M2 phenotype [162]. Bone marrow MSC (BMSC)-derived EVs deliver keratinocyte growth factor (KGF) mRNA to treat acute lung injury (ALI) caused by *E. coli* endotoxins [163]. Urinary exosomes, containing immune proteins like lysozyme C and myeloperoxidase, have been shown to effectively inhibit the growth of both pathogenic and commensal *E. coli* strains and induce bacterial lysis. This antibacterial activity is pH-dependent, being most effective at the acidic pH typical of human urine [164]. Meanwhile, biliary and intestinal epithelial EVs contain LL-37 and hBD-2, activating TLR-4 signaling to enhance antimicrobial defense [165]. A recent study showed that MXene-M2-Exo nanohybrids (FM-Exo) continuously release exosomes for up to 7 days, offering broad-spectrum antibacterial and immunosuppressive effects in a diabetic rat model [166]. In another study, host-derived small EVs from an infection contribute to the expansion of CD4+ T cells, leading to Th1-biased immune responses and this may serve as novel vaccine vectors against *Salmonella* and other Gram-negative bacteria [167]. In addition, EVs also help repair host cell membranes damaged by bacterial toxins and can act as decoys, binding and neutralizing toxins, whereby hEVs with ADAM10 protein can bind and neutralize *Staphylococcus aureus* alpha-toxin [168]. Outer membrane vesicles (OMVs) from *Burkholderia thailandensis* exhibited potent antibiofilm activity against *Streptococcus mutans*, reducing biofilm biomass. OMVs also acted synergistically with gentamicin, enhancing its activity against *S. mutans* biofilms [169]. EVs from *Mycobacterium tuberculosis*-infected macrophages carry bacterial RNA via the SecA2 secretion system. These EVs activate the host RIG-I/MAVS pathway, triggering type I IFN production and LC3-associated phagosome maturation, enhancing bacterial killing. Notably, EVs boost the efficacy of antibiotics like moxifloxacin, highlighting their potential as adjunct therapy for drug-resistant TB [170]. Similarly, *Lactobacillus*-derived EVs can modulate host gene expression and protect against vancomycin-resistant *Enterococcus faecium* infections [171]. Unlike traditional antibiotics, exosomes demonstrate a multi-targeted antibacterial effect, which reduces the likelihood of developing resistance. Table 6 summarizes antibacterial and antibiofilm activities of various EV types, their mechanisms of action, and potential applications.

Despite the promising potential of exosome-based treatments, further research is necessary to establish their safety and efficacy in clinical settings. While exosomes are naturally occurring, their capacity to carry a variety of bioactive molecules such as genetic material and toxins requires careful attention to exosome formulation and dosing to ensure safe and effective therapeutic use [172]. The storage stability of exosomes over extended periods remains a challenge [173]. Exosomes are susceptible to environmental stress, which may attenuate their therapeutic activity. Establishing protocols for the stability and preservation of exosome formulations is crucial for their successful translation into clinical applications [174]. Enhancing the delivery and targeting of exosomes to specific cells or tissues is another significant challenge. While exosomes inherently possess some targeting ability, their use in bacterial infections requires improved control over delivery mechanisms [175]. Strategies such as altering the surface of exosomes or incorporating specific targeting ligands may improve the specificity and efficiency of exosome-based therapies. Table 7 summarizes various sources of EVs and their respective attributes in terms of stability, therapeutic targeting, immune interaction, and key biomedical applications.

## 5. PDA Coatings and Exosomes in Antimicrobial Applications: A Synergistic Strategy

PDA coatings, especially when combined with EVs, offer versatile solutions for infection prevention, targeted therapy, and immune modulation. These strategies show promise for combating antibiotic-resistant bacteria, improving medical device safety, and advancing vaccine and diagnostic technologies. This coating could possess unique properties such as redox activity, strong surface adhesion, and rich chemical reactivity [176]. This coating not only enhances structural stability but also imparts antimicrobial functionalities through multiple mechanisms [177]. For instance, PDA-coated polypropylene meshes were reported to continuously generate H_2_O_2_ for over 48 h with intense bactericidal activity against both localized producers of ROS, which can serve as a non-antibiotic strategy against bacterial elimination [178]. This capability is particularly advantageous in infection-prone scenarios such as chronic wounds and medical implants, where long-lasting antibacterial effects are essential. PDA-coated exosomes can similarly act as localized sources of ROS, transforming them into self-sterilizing platforms capable of sustained antibacterial delivery without the need for additional chemical agents. This feature is particularly beneficial in infection-prone conditions, such as chronic wounds and implanted medical devices, where sustained antibacterial protection is critical. Coating exosomes with PDA can imbue them with localized ROS generation capacity, transforming them into self-sterilizing or antibacterial delivery systems without needing additional chemical agents [179].

In addition to ROS production, PDA coatings also significantly modify the surface characteristics of exosomes. These modifications, such as increased hydrophilicity, roughened surface, and the creation of a negative surface charge, serve to repel bacterial adhesion and preempt early biofilm formation [180]. Increased hydrophilicity reduces the tendency of bacteria to attach, while a roughened surface and negative charge create unfavorable conditions for bacterial colonization, thereby enhancing the exosomes’ resistance to infection and biofouling [181]. This anti-biofilm activity is particularly valuable in clinical applications like tissue engineering scaffolds and implanted devices, where bacterial colonization and biofilm formation are major causes of chronic infection and treatment failure.

PDA also supports extensive secondary functionalization. Its surface chemistry accommodates the immobilization of any number of bioactive agents such as AMPs, metal ions (e.g., Ag^+^, Cu^2+^), or targeting ligands without compromising exosome integrity [182]. Such versatility enables the development of multifunctional exosome-based drug delivery systems with the potential to accomplish cargo delivery and localized antimicrobial function in tandem. For instance, PDA-coated exosomes can be engineered to target antibiotic delivery to infected sites, thus achieving local therapeutic concentrations and minimizing systemic toxicity and resistance development.

Remarkably, PDA binds to EV membranes predominantly at the surface and not by disrupting the membrane. It forms a thin, conformal coating via covalent-like reactions such as Schiff–base formation and Michael addition with nucleophilic groups (amines, thiols) of membrane proteins, as well as non-covalent interactions such as hydrogen bonding, π–π stacking, and hydrophobic interactions with lipid molecules such as phosphatidylserine, cholesterol, and sphingolipids [183]. These interactions stabilize the exosome surface, provide a plastic interface for further functionalization, and maintain the structural and functional integrity of the vesicle [184].

This multifunctional interface allows for enhanced colloidal stability, PEGylation, ligand conjugation, and the incorporation of exosomes into sustained-release delivery vehicles. In biosensing applications, PDA coatings also facilitate detection signal reproducibility due to their stable interface with EV membranes [185]. PDA does not exhibit selective binding to specific lipid species, but EV lipid composition, such as phospholipids, cholesterol, and ceramides that regulate membrane curvature and PDA binding affinity, illustrates the contribution of membrane biophysics to coating effectiveness [186].

**Figure 2:**
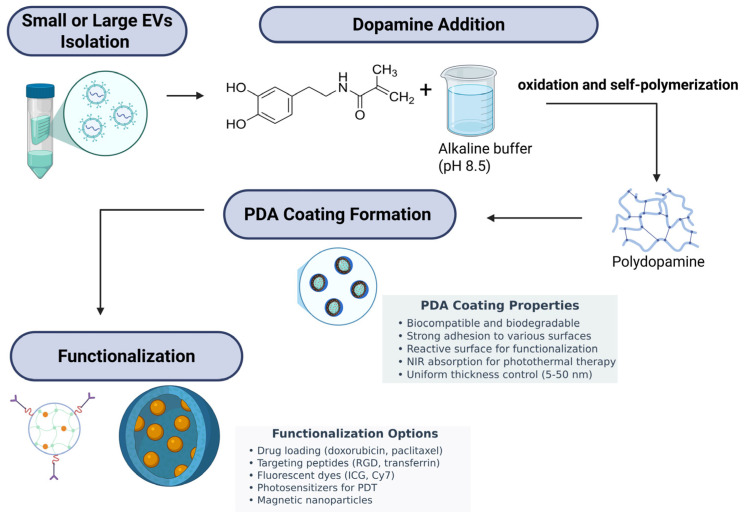
A schematic representation of the PDA coating of EVs.

**Figure 3:**
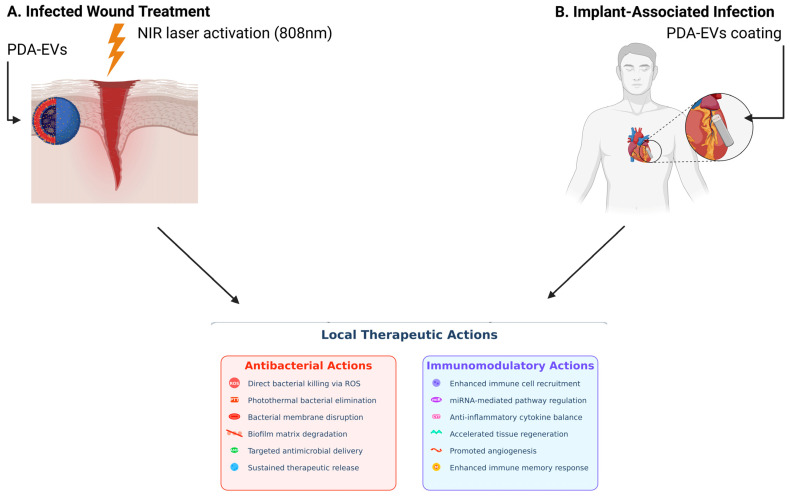
Therapeutic applications and mechanisms of PDA-exosome hybrids in antimicrobial treatment.

**Figure 4:**
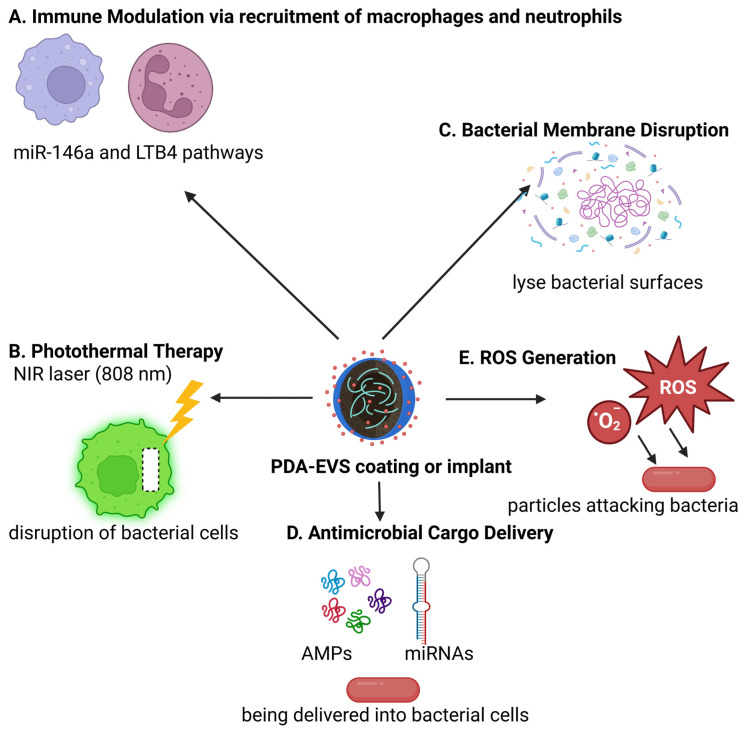
Antibacterial mechanisms of PDA-exosome hybrids.

Together, PDA-coated exosomes represent an antimicrobial therapy platform of the next generation with prolonged structural stability, intrinsic bactericidal effects via ROS generation, reduced bacterial colonization owing to anti-adhesive surface properties, and promise for biofunctional enhancements. They are highly promising in wound healing, protection of implantable devices, and infectious disease management in general. Continued research into their in vivo pharmacology, safety, and therapeutic utility will be required to best realize their clinical value. Figure 2 shows a schematic overview of the PDA coating strategy for EVs and their subsequent functionalization. Small or large EVs are first isolated and then mixed with dopamine in an alkaline buffer (pH 8.5), leading to dopamine oxidation and self-polymerization into PDA. This results in the spontaneous formation of a uniform PDA coating on the EV surface. The PDA layer, characterized by strong adhesion, biocompatibility, biodegradability, and reactive surface chemistry, serves as a versatile platform for further functionalization. Various functional moieties can be conjugated to the PDA-coated EVs, including therapeutic drugs (doxorubicin, paclitaxel), targeting peptides (RGD, transferrin), fluorescent dyes (ICG, Cy7), photosensitizers for photodynamic therapy (PDT), and magnetic nanoparticles. This multifunctional engineering enables enhanced targeting, imaging, and therapeutic efficacy for diverse biomedical applications. Figure 3 illustrates two clinical scenarios: (A) Infected wound treatment depicting a cross-section of skin layers (epidermis, dermis, subcutaneous) with a bacterial-infected wound cavity treated using a PDA-exosome hydrogel. Upon NIR laser activation, the PDA layer facilitates photothermal therapy (PTT) for bacterial clearance, while the exosomal cargo promotes tissue regeneration, angiogenesis, and immune modulation to accelerate healing. (B) Implant-associated infection showing a medical implant surrounded by biofilm-forming bacteria, where the PDA-exosome coating serves both preventive and therapeutic roles through biofilm disruption, antibacterial release, and immune regulation. The detailed mechanism panel highlights dual antibacterial actions (ROS generation, PTT, membrane disruption, biofilm degradation, antimicrobial peptide delivery, sustained release) and immunomodulatory effects (immune cell recruitment, miRNA delivery, cytokine modulation, tissue repair, angiogenesis, and immune memory enhancement). Figure 4 illustrates the key antibacterial actions of PDA-coated exosomes, including the following: (A) immune modulation via miR-146a and LTB4 pathways, (B) photothermal therapy (PTT) with NIR laser activation, (C) bacterial membrane disruption, (D) antimicrobial cargo delivery (AMPs and miRNAs), and (E) ROS generation for bacterial killing.

## 6. Advantages of Exosome-Encapsulated Antibiotics

Exosome-encapsulated antibiotics represent a breakthrough step beyond conventional antibiotics by attacking some of the most significant mechanisms of bacterial resistance. Their highest utility comes from their enhanced intracellular penetration to infection reservoirs. Many pathogens, such as *S. aureus* (including MRSA), often exist within host phagocytes such as macrophages, where conventional antibiotics are unpenetrated [187]. Exosomes, in contrast, have the capacity to accumulate in such immune cells effectively and colocalize with lysosomes, the intracellular location where bacteria are found delivering much greater intracellular levels of antibiotics like linezolid. Targeted delivery enables more efficient elimination of intracellular bacteria, eradicating reservoirs that are typically sites of chronic infection and resistance development [188]. In addition, exosome preparations maintain high intracellular concentrations of drug over long periods; for instance, studies have shown that concentrations of exosomal linezolid can reach as much as five-times that of unbound drug after 24 h [182]. This long-term exposure ensures that bacteria are exposed to bactericidal levels of the antibiotic for sufficiently long time periods to suppress survival and mutation-driven resistance mechanisms [189]. Exosomes are also endowed with low immunogenicity and reduced cytotoxicity, enabling multiple dosing without evoking adverse immune responses, preserving host immune integrity, and reducing the need for high systemic drug levels that are prone to favor resistant strain emergence [190]. Their bilayer lipid structure also allows for flexible drug loading, with the ability for hydrophilic and lipophilic encapsulation of drugs and potential for co-delivery of combination drugs such as antimicrobial peptides, immune modulators, or siRNAs against resistance [191]. Combination treatments enhance bactericidal efficacy while minimizing opportunities for the development of resistance. In addition, mannose-functionalized engineered exosomes may be engineered to preferentially target infected macrophages and accumulate in organs like the liver and spleen, where intracellular pathogens localize, thus increasing locoregional drug concentration while minimizing systemic exposure and its associated risks [192]. Although still in investigational stages, exosomes also possess the capability of penetrating and killing biofilms by having the ability to deliver active agents within these protected environments [193]. Combined, these sophisticated advantages render exosome-based antibiotic therapy a superior option to conventional therapies, not only by being able to reach further beyond the pharmacokinetic and pharmacodynamic limitations of free antibiotics but also by targeting the root of bacterial persistence and resistance [194]. Table 8 shows the comparative advantages of exosome-based nanotherapies over traditional antibiotics and unmodified exosome therapies in the context of antimicrobial and targeted infectious disease treatment.

Exosome nanotherapies are increasingly acknowledged to be promising biomedicine platforms due to their intrinsic biocompatibility, native targeting ability, and engineering potential. As naturally occurring extracellular vesicles, exosomes can be loaded with and target a wide range of therapeutic agents, such as antimicrobial drugs, with high specificity and minimal toxicity [195]. Such properties render exosomes excellent candidates for second-generation antimicrobial therapies in medical device sterilization, surgical prophylaxis, and infection treatment. In the application of medical devices, engineered exosomes can be used to deliver targeted antimicrobial agents to device surfaces, avoiding the toxic effects of conventional sterilizing agents like ethylene oxide or gamma radiation. When incorporated into hydrogels or nanocomposite coatings, exosomes provide extended antimicrobial release while maintaining a biocompatible interface, which reduces biofilm formation and inflammation risks associated with implants [196,197,198]. For prophylaxis during surgery, exosome products deliver antibiotics and immunomodulators specifically to the surgical site. MSC-derived exosomes are particularly recognized to have anti-inflammatory and immunoregulatory activities that can suppress post-surgery infection and restore tissue healing [199]. Their addition to wound dressings or surgical sealants imparts two-layered advantages such as the protection of microbial invasion with the simultaneous acceleration of regenerative processes such as angiogenesis and re-epithelialization [200]. In infection treatment, exosomes enable the efficient delivery of therapeutics into infected host cells beyond the principal shortage of traditional antibiotics. This is especially helpful for intracellular infections such as tuberculosis or MRSA [201]. Multifunctional payloads like RNA-based therapies or CRISPR-Cas9 can be packaged in exosomes and, in one delivery, allow for the combined eradiation of the pathogen and immune modulation. Repeated dosing is aided by low immunogenicity, which is ideal for chronic or relapsing disease [202]. In summary, exosome-based nanotherapies represent a paradigm shift approach to antimicrobial therapy, combining targeted delivery, enhanced safety, and multifunctional modality in one platform.

## 7. Other Promising Polymers for Antimicrobial Exosome Coatings

The modification of antimicrobial exosome coatings has emerged as a promising strategy to enhance their therapeutic efficacy, particularly in infection-prone diseases or combination therapies against microbial pathogens and host cells [203]. Among the most promising candidates are cationic polymers such as quaternized chitosan and polyethyleneimine (PEI), which exhibit potent antimicrobial activity through electrostatic binding with negatively charged bacterial membranes [204]. This interaction results in membrane destabilization, intracellular content leakage, and, ultimately, the death of the bacterial cell. Quaternized chitosan derivatives such as hydroxypropyltrimethyl ammonium chloride chitosan (HACC) are particularly known to form self-healing hydrogels that are capable of encapsulating exosomes, with the added antimicrobial protection and sustained release system [205]. PEI, a polycation that possesses superior antibacterial activity, is chemically constructible using highly advanced polymerization techniques like ATRP for modulating toxicity and maximizing biocompatibility [206]. The polymers are highly adaptable to direct exosome surface modification or incorporation in localized delivery vehicles, which support targeted antimicrobial activity while preserving exosomal cargo bioactivity.

Another essential category is poly(ethylene glycol) (PEG) and copolymers thereof, which, although not antimicrobial in nature, are vital for improving exosome circulation half-life and stability [207]. PEG forms a steric coating resistant to protein adsorption and immune cell recognition, thus prolonging the systemic half-life of exosomes. However, its failure to display antimicrobial activity necessitates its combination with other agents such as AMP, cationic polymers, or metal nanoparticles to produce a dual-functional coating [208]. For this, zwitterionic polymers are being considered alternative or additional options to PEG. Zwitterionic polymers, possessing balanced positive and negative charges, have highly hydrated, charge-balanced surfaces that resist nonspecific protein adsorption, bacterial adhesion, and biofilm formation [209]. Their antifouling properties, coupled with excellent biocompatibility, make them top candidates to be used for designing exosome coatings that will passively prevent microbial growth.

Polymer composites containing metal nanoparticles such as silver or copper have been remarkable in their attempts to further enhance antimicrobial activity. These nanocomposites possess bactericidal action resulting from the release of ions and ROS generation, which lead to irrecoverable damage to bacterial membranes and intracellular organelles [210]. These polymers are of particular interest with their wide-spectrum activity, such as drug-resistant bacterial strain effectiveness. Similarly, piperazine copolymers, which are a newer addition to antimicrobial materials, have strong membrane interaction and ROS-mediated antibacterial activity [211]. The polymers do not lose activity even after multiple cycles of water exposure and washing, making them suitable for permanent exosome coatings or scaffolds in regenerative medicine, where contamination by microbes is a concern.

Lastly, the application of 2D material–polymer composites, such as black phosphorus and molybdenum disulfide (MoS_2_), in exosome coatings is a cutting-edge approach towards antimicrobial enhancement [212]. They possess high surface areas, strong photothermal and oxidative activity, and inherent biocompatibility. Upon hybridization with polymers, they enable the creation of multifunctional coatings that not only combat microbial infections through ROS production and membrane disruption but also preserve or enhance the biological delivery function of exosomes [213]. Although largely studied in the framework of biomedical implants and wound dressings, with a synergy of biocompatibility and antimicrobial activity, these materials enable a multifunctional yet adjustable platform to enable both the security and therapeutic application of exosome-based therapies, especially in those instances where microbial infection or co-infection is a clinical barrier. Table 9 presents comparisons between various coating materials commonly employed to functionalize exosomes for antimicrobial purposes.

## 8. Challenges in Applying Antimicrobial Coatings to Exosomes

The application of antimicrobial polymer coatings on exosomes presents a new avenue for combat against bacterial infection, particularly drug-resistant bacteria. However, the approach is under special challenges owing to the sensitivity and complex structure of exosomes. Exosomes are nanoscale extracellular vesicles enclosed in a lipid bilayer, containing functional proteins, lipids, and nucleic acids and are vital to their roles as carriers of intercellular communication, immune modulation, and therapy. A major challenge is maintaining the structural integrity and biological functions of these vesicles intact during coating. A number of surface proteins on exosomes [214], for example, CD47, are crucial in the immune evasion and targeting of recipient cells effectively. Hostile coating conditions or reactive polymers may destroy the membrane integrity or occlude such surface labels, eliminating the therapeutic advantage of the exosomes [215].

Another challenge is concerned with the best balance between antimicrobial activity and biocompatibility. Most cationic polymers such as polyethyleneimine (PEI) or quaternized derivatives of chitosan function through disrupting bacterial membranes [216]. Even as effective at destroying microbes, such processes can prove harmful to exosomes themselves as well as to host cells unless the coatings are precisely tuned. Too harshly acting coatings would kill the membrane of the exosome or initiate cytotoxic and inflammatory responses on injection [217]. Therefore, the development of coatings that maintain high antimicrobial activity and minimize bystander damage is a main research focus. This involves polymers that are selectively bactericidal, biodegradable, and non-toxic at therapeutic concentrations, along with better methods that maintain exosome integrity [218].

Technical challenges also include the deposition of uniform, stable coatings on such small and heterogeneous particles. Exosomes are 30–150 nm in diameter and exhibit significant heterogeneity in physical properties and surface chemistry [219]. Balanced polymer deposition within this size is not an easy feat, and asymmetric coatings may affect colloidal stability, biodistribution, and therapeutic efficacy. In addition, coating procedures have the potential to inadvertently contain contaminants or induce exosome aggregation, both of which compromise the purity, safety, and reproducibility required in clinical applications [220]. Scalable and reproducible methods for polymer coatings that assure monodispersity and retain functional cargo are not yet available and represent a significant bottleneck for industrial translation [221].

The biological heterogeneity of the exosome adds another layer of complexity. Exosome subpopulations may differ in their cargo, surface antigens, and targeting capabilities, such that a single coating procedure might not be optimally effective across the population of exosomes [222]. Moreover, the influence of polymer coatings on the pharmacokinetics and biodistribution of exosomes is still not well understood. The modulation of surface properties can impact interactions with serum proteins, immune cells, or off-target tissues, with risks of immunogenicity or off-target effects [223]. These concerns emphasize the need for the rigorous characterization of coated exosomes and thorough evaluation of their safety profiles in preclinical models.

Finally, the biggest barrier to large-scale deployment is that no universally adopted protocols for exosome isolation, purification, and surface modification exist [224]. Variation in isolation protocols, such as ultracentrifugation, size-exclusion chromatography, or precipitation kits, creates variability in yield and purity that makes downstream coating and analysis difficult [225]. Without a consensus of best practices and quality control procedures, it is difficult to compare study findings, and regulatory approval becomes harder to achieve. For clinical translation, robust manufacturing processes ensuring batch-to-batch reproducibility, scalability, and regulatory acceptance are a necessity.

## 9. Translational Challenges and Regulatory Hurdles of Exosome-Based Nanotherapies

Exosome nanotherapies are a highly promising class of therapy delivery platforms owing to their intrinsic biocompatibility, negligible immunogenicity, and ability to bypass biological barriers but remain restrained by a myriad of interrelated translational as well as regulatory challenges in clinical translation [226]. Among the principal challenges is that there are no available scalable and reproducible manufacture processes. Although techniques like ultracentrifugation, size-exclusion chromatography, and precipitation are widely employed, they are marred by low yield, non-vesicular material contamination, and low throughput, rendering them unsuitable for clinical-scale manufacture [227]. Hybrid exosomes with synthetic liposomes and native vesicles are a potential solution to these limitations in scalability and cross-loading versatility, although their long-term stability, immunocompatibility, and therapeutic homogeneity are not yet sufficiently studied [228]. High drug loading is another significant challenge, particularly for hydrophilic biomolecules such as proteins and nucleic acids, which face difficulty in lipid bilayer crossing. Even though methods like electroporation, sonication, and nanoporation increase loading efficacy for RNA therapies, they compromise the integrity and reproducibility of exosomes, making stoichiometric and localization control over the cargo complex and uncertain [229]. Stability and storage are other concerns; the most commonly used technique, cryopreservation, can induce aggregation and loss of functional properties, and although more recently developed protocols like lyophilization and microneedle patch delivery seem to help preserve exosome integrity and enhance tissue retention, standardized long-term storage procedures such as for liposomal drugs remain underdeveloped [230]. In addition, the inherent heterogeneity of exosomes such as various sizes, surface antigens, and bioactive contents depending on their cell source and physiological condition causes difficulty in the delivery of uniform therapeutic effects and the implementation of universal characterization protocols [231]. The absence of approved tests of such key parameters as particle size, zeta potential, protein markers, and potency severely limits inter-laboratory reproducibility and impedes qualification by regulators [232]. Regulator-wise, exosome-based therapies fall in a regulatory limbo between nanomedicines and biologics and consequently face unclear classification and regulation, with regulatory agencies like the FDA and EMA to date having not prepared clear regulatory guidelines [233]. The heterogeneity and complexity of exosomal cargo also complicate demonstrating safety and efficacy, with the potential to induce immunogenicity, off-target toxicity, and tumor growth-promoting capabilities by some exosome populations, most notably cancer cell-derived ones [234]. Furthermore, the lack of GMP-grade manufacturing processes and inconsistent quality control protocols on the purity, sterility, and elimination of contaminants is the most significant barrier to clinical approval. Even in the face of several initial-stage clinical trials testing exosome-based treatments for cancers such as melanoma and acute respiratory distress syndrome that are ongoing, none have yet resulted in regulatory approval, emphasizing a concern for proper trial design, uniform endpoints, and robust long-term safety information [235]. Sealing these translational gaps will require coordinated interdisciplinary effort with progress in microfluidic-based isolation technologies, exosome engineering with exquisitely defined targeting ligands, and collaborative ventures between academia, industry, and regulators to establish clear guidelines and standard operating procedures [236]. In conclusion, as thrilling as the therapeutic potential of exosome nanotherapies is, it will be critical to bridge the existing translational and regulatory hurdles through standardization, scalable manufacturing, clinical rigor, and worldwide harmonization in a bid to actualize their complete clinical potential.

## 10. Conclusions

AMR presents a pressing global health crisis that demands innovative, multifaceted therapeutic strategies. This review highlights the promising synergy between PDA, a broad-spectrum, bioinspired polymer, and EVs for antimicrobial applications. PDA provides inherent antibacterial activity through ROS generation, photothermal effects, and strong adhesion, while exosomes offer natural targeting, intercellular communication, and the efficient delivery of antimicrobial peptides and other therapeutic cargo. The combination of PDA and exosomes yields a hybrid nanoplatform with enhanced stability, surface modifiability, and localized antibacterial action, suitable for applications such as wound healing and implant-associated infections. Several future directions support the advancement of this field. These include the integration of AMPs into polymer-coated exosomes via stable immobilization or controlled release for effective and biocompatible bacterial killing. Stimuli-responsive coatings that activate in response to pH, ROS, or bacterial enzymes enable targeted antimicrobial action. Zwitterionic polymers, as alternatives to PEG, offer improved biocompatibility and reduced immunogenicity while maintaining stealth properties. Modular and clickable polymer coatings, including layer-by-layer assemblies, allow for tunable combinations of antifouling, targeting, antimicrobial, and self-healing functions. Additionally, incorporating nanomaterials like black phosphorus or metal nanoparticles enhances antimicrobial potency through synergistic ROS generation and membrane disruption. Bioinspired coatings, such as PDA itself, provide universal adhesion and customizable surfaces for ligand or drug attachment. The emergence of hybrid exosome-mimetic vesicles, synthetic systems mimicking natural exosomes with improved engineering control offers promise for scalable, reproducible applications. Ultimately, clinical translation will depend on standardized manufacturing, realistic infection models, and regulatory alignment to ensure safety, efficacy, and consistency.

## Figures and Tables

**Table 1:** The mechanisms of antibiotic resistance.

Mechanism	Description	Examples	Clinical Relevance
Efflux Pumps	Active transport proteins that expel antibiotics out of bacterial cells.	AcrAB-TolC (*E. coli*), MexAB-OprM (*P. aeruginosa*)	Multidrug resistance; overexpression in UPEC and *P. aeruginosa* leads to resistance to β-lactams, fluoroquinolones, etc.
Enzymatic Inactivation	Production of enzymes that degrade or modify antibiotics, rendering them inactive.	β-lactamases (classes A–D), aminoglycoside-modifying enzymes [AAC(6′)-Ib], macrolide esterases, TetX, ADP-ribosyltransferases	Key mechanism in resistance to β-lactams, aminoglycosides, macrolides, rifamycins, etc.
Target Site Alteration	Modification of antibiotic targets to reduce or abolish drug binding.	rpoB (rifamycin resistance), gyrA/parC (fluoroquinolones), mecA (MRSA), PBP modifications (*S. pneumoniae*), 23S/16S rRNA methylation	Resistance in M. tuberculosis, *S. aureus*, *S. pneumoniae*; major contributor to therapeutic failure.
Reduced Membrane Permeability	Altered outer membrane composition or porin expression, limiting drug entry.	OprD (*P. aeruginosa*), OmpK36 (*K. pneumoniae*), OmpF (*E. coli*)	Reduces susceptibility to β-lactams, quinolones, and other hydrophilic antibiotics in Gram-negative bacteria.
Biofilm Formation	Structured communities embedded in an extracellular matrix that restricts antibiotic penetration and enhances tolerance.	*S. epidermidis*, *K. pneumoniae*, *P. aeruginosa* (CF patients), *S. aureus*, CoNS	Biofilms increase resistance 10–1000×; associated with chronic infections and medical device colonization.
Immune Evasion	Strategies to avoid host immune responses and prolong infection, allowing more time for resistance to develop.	Induction of IL-10, TGF-β; intracellular survival; LPS/capsule modification; biofilm-mediated immune evasion	Enables chronic infection and persistence; enhances time for resistance trait selection and maintenance.

**Table 2:** Emerging antimicrobial strategies to combat drug-resistant pathogens.

Strategy	Mechanism/Description	Examples	Potential Benefits
Exosome-Based Drug Delivery	Natural vesicles deliver antimicrobial agents directly to target cells.	MSC-derived exosomes carrying antibiotics or siRNA.	Targeted delivery, reduced toxicity, immune evasion.
CRISPR-Cas Antimicrobials	Gene editing tools used to disrupt resistance genes in bacteria.	CRISPR-Cas9 targeting *blaNDM*, *mecA* genes.	Specific targeting of resistance genes; prevents horizontal gene transfer.
Quorum Sensing Inhibitors (QSIs)	Block bacterial communication to prevent biofilm formation and virulence.	Furanones, AHL analogs.	Disarm pathogens without killing, reducing selective pressure.
Phage Therapy	Use of bacteriophages to infect and lyse resistant bacteria.	*Listeria*-specific phages in food safety; *Pseudomonas* phages in lung infections.	Host-specific, can co-evolve with bacteria, minimal dysbiosis.
Nanoparticles	Nanomaterials with inherent antimicrobial properties or used as drug carriers.	Silver nanoparticles, liposomes, polymeric NPs.	Enhanced penetration, controlled release, membrane disruption.
Synthetic Polymers	Engineered molecules for targeted delivery or direct antimicrobial action.	Cationic polymers, dendrimers, polymer-drug conjugates.	Broad-spectrum activity, biofilm penetration, reduced resistance development.
Engineered Probiotics	Genetically modified microbes that detect and kill resistant pathogens.	*Lactobacillus* strains producing bacteriocins or CRISPR systems.	Gut microbiome protection, pathogen-specific killing.
Microbiota-Based Interventions	Use of beneficial microbes to outcompete or modulate pathogens.	Fecal microbiota transplantation (FMT), synbiotics.	Restore healthy microbiota, indirect suppression of resistance.
Combination Therapy	Use of multiple agents to target different resistance mechanisms.	Colistin + rifampin for MDR *Acinetobacter*; β-lactam + β-lactamase inhibitor.	Synergistic effects, delayed resistance emergence.

**Table 3:** Key properties and functional applications of polydopamine (PDA).

Category	Specific Property	Functional Implications/Applications
Adhesion	Strong, universal adhesion to diverse substrates	Surface coating, implant modification, wet-interface applications
	Adhesion in aqueous environments	Biomedical use (e.g., tissue contact, wound dressings)
Chemical Reactivity	Catechol, amine, imine groups	Covalent bonding, surface engineering, biomolecule immobilization
	Quinone formation via catechol oxidation	Metal chelation, redox activity, cross-linking, catalytic functionality
	Michael addition/Schiff base reactivity	Conjugation with thiols, amines, hydroxyls
Biocompatibility	Non-toxic, supports cell adhesion and proliferation	Tissue engineering, drug delivery, biosensors
Morphology & Structure	Tunable to nanoparticles, films, capsules, core–shell	Nanoplatforms for imaging, targeting, catalysis
Solubility	Water-insoluble (bulk), organic-solvent soluble (nano)	Organic-phase processing; solubility enhanced by PEGylation
	Enhanced colloidal stability via surface modification	Long-term physiological stability
Bioactivity	ROS generation (e.g., H_2_O_2_, superoxide)	Antibacterial, anticancer therapy (requires control for safety)
	Membrane disruption	Antimicrobial coatings
Photothermal Properties	NIR light-to-heat conversion	Photothermal therapy (PTT) for cancer and infections
Electrochemical Behavior	Redox-active; electron donor under stimuli	Biosensors, energy devices, electrocatalysis
Catalysis	Intrinsic and synergistic with nanoparticles (e.g., Ag)	Environmental cleanup, antibacterial agents, smart nanomaterials

**Table 4:** Summary of antimicrobial mechanisms and synergistic applications of PDA.

Strategy/Mechanism	Description	Examples	Antimicrobial Benefits
ROS Generation	Catechol oxidation generates H_2_O_2_ and superoxide radicals	PDA thin films, PDA-metal hybrids (e.g., PDA-Ag)	Oxidative damage to membranes, proteins, and DNA
Electrostatic Membrane Disruption	Protonated amine groups interact with bacterial membranes	PDA nanoparticles with polyarginine	Membrane lysis and bacterial death
Surface Roughness Enhancement	rPDA coatings improve contact and mechanical disruption	Rough PDA coatings on surfaces	Higher bacterial adhesion and enhanced killing compared to smooth PDA
Metal Chelation and Hybridization	Catechol groups chelate metals for synergistic killing	PDA-Ag NPs, PDA-Au NPs, PDA–metal–organic frameworks	Sustained release of ions, increased ROS, prolonged antimicrobial action
Photothermal Therapy (PTT)	PDA absorbs NIR light and converts it into localized heat	PDA-ZIF-8 nanostructures, PDA-based microneedles, PDA-Polymyxin B	Thermal denaturation of bacterial proteins and biofilm disruption
Photodynamic Therapy (PDT)	Light-activated ROS generation using PDA’s redox properties	PDA-MOFs, PDA-curcumin, PDA/Fe_3_O_4_ nanozymes	Light-induced oxidative stress leads to bacterial death
N-Halamine Functionalization	Chlorinated amine groups release halide species	PDA/PEI chlorinated films, halamine-modified PDA coatings	Oxidative damage to bacterial enzymes and proteins, durable and repeatable killing
Antimicrobial Peptide (AMP) Immobilization	Covalent or non-covalent attachment of AMPs	PDA-KR-12, PDA-CWR11, PDA-lipopeptide (SL1.15), PDA-Mel4	Localized, sustained antimicrobial action, minimal toxicity, biofilm inhibition
Peptidomimetic Conjugation	PDA facilitates binding of synthetic antimicrobial peptides	PDA-coated surfaces with RK758, Melimine, etc.	Broad-spectrum antimicrobial action, biofilm prevention
Antibiotic Loading and Controlled Release	PDA enables sustained antibiotic delivery	PDA-GO-tetracycline–Ag composites, PDA-Ag@Gen vs. Ag/Gen@PDA	Prolonged antimicrobial action, reduced burst release, biofilm suppression
Enzyme Immobilization	PDA coatings stabilize antimicrobial enzymes	PDA-lysostaphin functionalized surfaces	Enzyme-mediated bacterial lysis, preserved enzymatic activity
Synergistic Hybrid Nanoplatforms	Combined strategies for enhanced effects	PDA-TiO_2_ (photocatalytic), PDA-AuNPs (delivery + dispersion), PDA-PEI–Polymyxin B + PTT	Multifunctional action: targeting, penetration, ROS, PTT, and sustained delivery

**Table 5:** Comparative overview of different types of extracellular vesicles.

Feature	Small EVs (Exosomes)	Large EVs (Microvesicles)
Size Range	~30–150 nm	~100 nm to >1 µm
Biogenesis Origin	Endosomal pathway (MVBs/ILVs)	Plasma membrane budding and fission
Key Formation Process	Endocytosis → Early/late endosomes → ILVs → MVBs → Exocytosis	Direct outward budding from plasma membrane
Mechanisms Involved	ESCRT-dependent and ESCRT-independent (e.g., ceramide, tetraspanins)	Cytoskeletal rearrangement, calcium signaling, ARF6, RhoA, calpain
Surface Markers	CD9, CD63, CD81, ALIX, TSG101	Phosphatidylserine (detected by annexin A5/A1), integrins, CD40, ARF6
Cargo Composition	Proteins, lipids, miRNAs, mRNAs, lncRNAs, metabolites	Cytosolic proteins, membrane proteins, organelle fragments
Release Mechanism	Fusion of MVB with plasma membrane (Rab GTPases, SNAREs)	Budding and shedding from cell surface
Interaction with Target Cells	Endocytosis, membrane fusion, or ligand–receptor interaction	Same as exosomes, depending on content and surface molecules
Terminology (MISEV2023)	Preferred functional classification; biogenesis-based terms used if justified	Same; shift toward functional/operational definitions
Function	Intercellular communication, immune modulation, angiogenesis, metastasis	Cell signaling, coagulation, inflammation, immune response

**Table 6:** Antibacterial and antibiofilm properties of EVs from various sources.

EV Source	Antibacterial Target	Mechanism of Action	Applications
Bovine milk-derived exosomes	MDR bacteria	Deliver isobavachalcone and polymyxin B; 99% bacterial elimination	Wound healing, food preservation
Honey-derived EVs (HEc-EVs)	*Streptococcus mutans*	Contain MRJP1, defensin-1, jellein-3; disrupt biofilm	Oral health, natural antimicrobial agent
Pasteurized cow’s milk exosomes	*Staphylococcus aureus*	Dose-dependent growth inhibition; delayed lag/generation time	Food safety
Camel milk-derived exosomes (CM-EXOs)	*Escherichia coli*	Bacteriostatic; Gram-negative specificity	Animal health
Bovine colostrum-derived exosomes	*Staphylococcus aureus*	Disrupt oxidative phosphorylation; reduce ATP	Infection control
MSC-derived exosomes	Gram+ and Gram− bacteria	AMPs (e.g., beta-defensin-2), activate TLR-4, enhance macrophage/neutrophil activity	Tissue repair, infection therapy
BMSC-derived EVs	*E. coli*-induced ALI	Deliver KGF mRNA for tissue regeneration	Acute lung injury therapy
Urinary exosomes	*E. coli* (pathogenic and commensal)	Lysozyme C and myeloperoxidase; pH-dependent lysis	Urogenital infection defense
Biliary/Intestinal epithelial EVs	Broad spectrum	Contain LL-37, hBD-2; TLR-4 activation	Gastrointestinal immunity
MXene-M2-Exo (FM-Exo)	Broad spectrum (diabetic wound)	Sustained exosome release; antibacterial and immunosuppressive	Diabetic wound healing
Infection-derived host EVs	*Salmonella*, Gram-negative bacteria	Expand CD4+ T cells; induce Th1-biased response	Vaccine development
Host EVs (with ADAM10)	*Staphylococcus aureus* alpha-toxin	Bind/neutralize bacterial toxins	Antitoxin strategy
OMVs (*Burkholderia thailandensis*)	*S. mutans*	Antibiofilm; synergize with gentamicin	Biofilm-targeted therapy
EVs from *M. tuberculosis*-infected macrophages	*M. tuberculosis*	Deliver bacterial RNA via SecA2; trigger RIG-I/MAVS and LC3-associated phagosome maturation	TB adjunct therapy
*Lactobacillus*-derived EVs	*VRE faecium*	Host gene modulation; protection against infection	Probiotic therapy, resistance control

**Table 7:** EVs sources: stability, targeting, and clinical applications.

EV Source	Stability and Processing	Targeting and Therapeutic Potential	Immune Interaction and Safety	Key Applications
Bovine Milk-Derived Exosomes	Stable; affected by industrial processing	Drug delivery, tumor targeting	Cross-species tolerance; safe oral use	Cancer therapy, oral drug delivery
Honey-Derived EVs (HEc-EVs)	Stable; <150 nm size	Antibacterial, antibiofilm	Antimicrobial peptides; biofilm modulation	Dental caries prevention
Pasteurized Cow’s Milk Exosomes	Partial preservation of bioactive cargo	Similar to milk exosomes	Some immune proteins preserved	Nutritional and therapeutic supplements
Camel Milk-Derived Exosomes	Stable; unique cargo	Anticancer, antioxidant, anti-inflammatory	Immunomodulatory via lactoferrin and casein	Cancer, inflammation, oxidative stress
Bovine Colostrum-Derived Exosomes	Stable; rich in growth factors	Hair regeneration, tissue repair	Safe with minimal adverse effects	Hair loss, wound healing
MSC-Derived Exosomes	Stable; modifiable	Immunomodulation, anti-inflammatory, tissue repair	Low immunogenicity; promote immune tolerance	Autoimmune diseases, inflammation, cancer
BMSC-Derived EVs	Stable; miRNA-rich	Anti-fibrotic, anti-inflammatory	Modulate inflammatory cytokines	Fibrotic skin diseases
Urinary Exosomes	Stable with protease inhibitors	Diagnostic biomarkers	Low immune activation	Kidney/urinary diseases biomarkers
Biliary/Intestinal Epithelial EVs	Limited data	Gut immunity and homeostasis	Likely immune-modulatory	Gut health
MXene-M2-Exo (FM-Exo)	Emerging technology	Enhanced delivery and imaging	Under investigation	Nanomedicine
Infection-Derived Host EVs	Variable	Modulate infection and immunity	Influence pathogen-host interactions	Infectious disease research
OMVs (*Burkholderia thailandensis*)	Bacterial vesicles	Immune modulation, vaccine potential	Can trigger immune responses	Vaccine development, pathogenesis
EVs from *M. tuberculosis*-Infected Macrophages	Host-pathogen interaction vesicles	Biomarkers, immune modulation	Influence tuberculosis immunity	TB diagnosis and therapy
*Lactobacillus*-Derived EVs	Stable; probiotic origin	Immune modulation, gut homeostasis	Promote mucosal immunity	IBD, gut health

**Table 8:** Summary of distinctive attributes of antimicrobial delivery strategies.

Aspect	Conventional Antibiotics	Standard Exosome Therapy	Exosome-Based Nanotherapies
Intracellular Delivery	Limited penetration into intracellular pathogens	Natural biodistribution, moderate targeting	Enhanced intracellular delivery via engineering
Efficacy	Variable, often limited by resistance and bioavailability	Effective in immune modulation and cargo delivery	Superior efficacy in intracellular infections and targeted therapy
Immunogenicity	Potentially high with systemic toxicity	Low immunogenicity	Very low immunogenicity, reduced toxicity
Cargo Versatility	Mostly small molecules	Proteins, nucleic acids, small molecules	Broad cargo loading (hydrophilic/lipophilic drugs, proteins, RNA)
Stability	Variable, often requires formulation	Stable, biocompatible	High stability, modifiable for enhanced targeting

**Table 9:** Comparative review of hybrid and polymer coatings for antimicrobial exosome use.

Polymer/Hybrid	Antimicrobial Mechanism	ROS Generation	Coating Stability	Anti-Biofilm Effect	Functionalization Capacity
PDA (Polydopamine)	Sustained ROS (H_2_O_2_), surface charge repulsion	High (continuous)	Strong (catechol adhesion)	Strong	High (binds AMPs, metals, drugs)
PEG (Polyethylene Glycol)	Stealth coating; not inherently antimicrobial	None	Moderate (steric repulsion)	Weak	Moderate (limited active binding)
Quaternized Chitosan	Electrostatic bacterial membrane disruption	Low	Moderate (pH-sensitive)	Moderate	High (polyamine-rich structure)
PEI (Polyethyleneimine)	Strong membrane interaction; electrostatic killing	Moderate	Strong (polymeric film)	Moderate	High (modular chemical backbone)
Zwitterionic Polymers	Antifouling via hydration layer; passive microbial inhibition	None	Strong (hydrated layer)	Strong	Low–moderate
Metal NP Composites	Ion release, ROS, membrane damage	High	Variable (matrix-dependent)	Strong	Moderate–high
2D Material Hybrids (e.g., MoS_2_)	Photothermal killing, ROS, high surface area	High	High	Strong	High (multifunctional surface)

## Data Availability

There are no data to support the findings of this review.
